# Genome Sequencing of a Novel Coronavirus SARS-CoV-2 Isolate from Iraq

**DOI:** 10.1128/MRA.01316-20

**Published:** 2021-01-28

**Authors:** Nihad A. M. Al-Rashedi, Danilo Licastro, Sreejith Rajasekharan, Simeone Dal Monego, Alessandro Marcello, Murad G. Munahi, Basel Saber Odda, Yasir Adil Jabbar Alabdali, Laith A. H. ALObaidi, Ali Jasim, Ibrahem A. Abdulzahra, Karar Kadhim, Ali Awad, Mohamed Bachay

**Affiliations:** a Department of Biology, College of Science, Al-Muthanna University, Samawah, Iraq; b RGO Open Lab Platform for Genome Sequencing, Trieste, Italy; c Laboratory of Molecular Virology, International Centre for Genetic Engineering and Biotechnology (ICGEB), Trieste, Italy; d Department of Chemistry, College of Science, Al-Muthanna University, Samawah, Iraq; e Department of Public Health, Al-Muthanna Health Directorate, Samawah, Iraq; Queens College

## Abstract

The coding-complete genome sequence of a severe acute respiratory syndrome coronavirus 2 (SARS-CoV-2) strain isolated from an Iraqi patient was sequenced for the first-time using Illumina MiSeq technology. There was a D614G mutation in the spike protein-coding sequence. This report is valuable for better understanding the spread of the virus in Iraq.

## ANNOUNCEMENT

In December 2019, severe acute respiratorysyndrome coronavirus 2 (SARS-CoV-2) emerged in Wuhan, China. This virus belongs to the *Betacoronavirus* genus of the *Coronaviridae* family (1). RNA viruses are characterized by a high rate of genetic mutations, which enables them to escape host defenses and may also affect the discovery of an effective vaccine and the status of reverse transcription-quantitative PCR (RT-qPCR) coronavirus disease 2019 (COVID-19) detection (2, 3). Therefore, identifying the SARS-CoV-2 genome sequences and understanding their mutations are important. Here, we sequenced the coding-complete genome of a SARS-CoV-2 strain from an Iraqi patient.

A nasopharyngeal swab was collected from a patient with mild symptoms (26-year-old female infected in Samawah, Iraq) and added to Accuzol (Bioneer, Taejeon, South Korea) at a 1:3 (vol/vol) ratio. Viral RNA was extracted following the manufacturer’s protocol. Briefly, the pellet was resuspended in RNase-free water, and the RNA sample was concentrated using the RNA Clean and Concentrator kit (Zymo Research, CA, USA). The presence of SARS-CoV-2 RNA was confirmed using a Luna universal probe one-step RT-qPCR kit (NEB, MA, USA) and a primer/probe targeting the nucleocapsid gene (4), and viral RNA quantification was conducted using an *in vitro*-transcribed RNA standard (5). A Qubit 2.0 fluorometer (Thermo Fisher Scientific, MA, USA) and Agilent 2100 Bioanalyzer (Agilent Technologies, CA, USA) were used to assess the RNA quantity and quality. From the total RNA, 100 ng was processed using a Swift Amplicon SARS-CoV-2 research panel (Swift Biosciences, USA). The library obtained passed a quality check and was quantified before being used at equimolar concentrations. High-throughput sequencing was conducted using an Illumina MiSeq sequencer following the standard procedure. The read length was 150 bp. This produced 1,222,270 reads. The raw sequence data were quality controlled using FastQC v0.11.9 (https://www.bioinformatics.babraham.ac.uk/projects/fastqc/). Genome assembly was conducted using MEGAHIT v1.2.9 (6), and the genome size was 29,720 bp with a coverage depth of 5,340× (SAMtools v1.9) (7) and an overall GC content of 38.01%. This study was approved by the Research Ethics Committee at the University of Al-Muthanna (2529sci on 26 April 2020). Mapping of the obtained strain, hCoV-19/Iraq/ICGEB-5T (GISAID accession number EPI_ISL_582030), was accomplished using CoVsurver in GISAID (8), and the Wuhan strain (BetaCoV/Wuhan/WIV04/2019) was used as a reference. Analysis of this strain revealed eight mutations. These mutations were accompanied by four amino acid changes (Table 1). Among these changes, the following two infrequent amino acid changes were observed: NSP8-S76F, which has already been identified in three countries, and N-G215S, which has been identified in 10 countries to date. To estimate the effect of these amino acid changes on the stability of NSP8 and N proteins, the online server DUET (9) was used; it was predicted that these proteins are destabilized by the reported amino acid changes. Default parameters were selected for all the software and tools used in this work.

**TABLE 1:** Genome features of the SARS-CoV-2 isolate in Iraq

Isolate name	Sequence length (bp)	No. of mutations	Mutations	GISAID clade	Nextstrain clade
hCoV-19/Iraq/ICGEB-5T	29,720	8	C241T, C3037T, C10078T, C12318T, C18877T, A23403G, G25563T, G28916A	GH	20A

**FIG 1:**
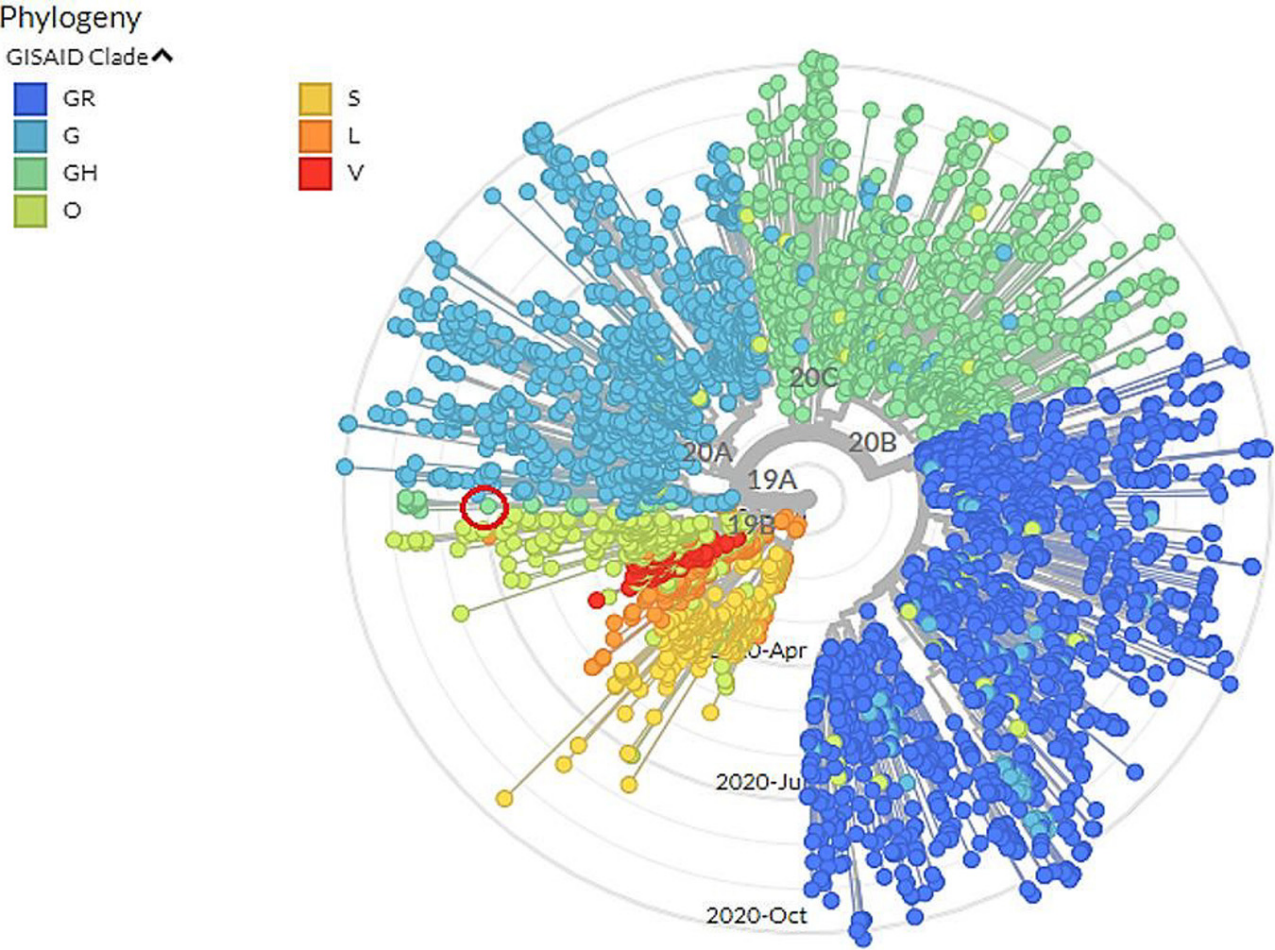
Phylogenetic tree of SARS-CoV-2 strains, including an isolate from Iraq (June 2020) as depicted in the open red circle (GISAID accession number EPI_ISL_582030).

The Nextclade v0.8.1 Web tool (10) was employed to generate a phylogenetic tree and a clade assignment. The obtained phylogenetic tree was rooted with strains from Wuhan, China. The phylogenetic tree revealed that strain hCoV-19/Iraq/ICGEB-5T belongs to the Nextstrain clade 20A and GISAID clade GH (Fig. 1). However, the GH clade is much more prevalent in Europe and North America (11–13).

### Data availability.

The coding-complete genome sequence of hCoV-19/Iraq/ICGEB-5T was deposited under GenBank accession number MW290973 and SRA accession number PRJNA689203 (BioProject). The GISAID accession number for hCoV-19/Iraq/ICGEB-5T is EPI_ISL_582030.
